# The relationship between absolute counts of lymphocyte subsets and clinical features in patients with pulmonary tuberculosis

**DOI:** 10.1111/crj.13490

**Published:** 2022-05-06

**Authors:** Hui‐Ru An, Xue‐Juan Bai, Jian‐Qin Liang, Tao Wang, Zhong‐Yuan Wang, Yong Xue, Yin‐Ping Liu, Lan Wang, Xue‐Qiong Wu

**Affiliations:** ^1^ Department of Tuberculosis, Senior Department of Tuberculosis The Eighth Medical Center of PLA General Hospital Beijing China; ^2^ Tuberculosis Prevention and Control Key Laboratory, Beijing Key Laboratory of New Techniques of Tuberculosis Diagnosis and Treatment, Institute for Tuberculosis Research, Senior Department of Tuberculosis 8th Medical Center of Chinese PLA General Hospital in China Beijing China

**Keywords:** absolute counts, flow cytometry, immunity, lymphocyte subsets, pulmonary tuberculosis

## Abstract

**Background:**

The aim of the present study is to investigate the clinical value and characteristics of peripheral blood lymphocyte subsets in patients with pulmonary tuberculosis (PTB) using flow cytometry.

**Methods:**

The absolute counts of T, CD4^+^T, CD8^+^T, natural killer (NK), NKT and B lymphocytes in 217 cases of PTB were detected, and the variations in lymphocyte subset counts between different ages and genders and between aetiological detection results and chest radiography results were analysed.

**Results:**

In 75.3% of the patients with PTB, six subset counts were lower than the normal reference range, and 44% showed lower‐than‐normal CD4^+^T lymphocyte levels. The counts of T, CD4^+^T, CD8^+^T and B lymphocytes were significantly lower in patients aged >60 years, and the NKT cell counts were significantly lower in female patients than in male patients. Among the patients with positive aetiological results, 40.8% had reduced CD8^+^T counts; these were significantly lower than those in patients with negative aetiological results (*P* = 0.0295). The cell counts of T, CD4^+^T, CD8^+^T and B lymphocytes reduced as lesion lobe numbers increased. The counts of T, CD4^+^T and CD8^+^T lymphocytes were significantly higher in the group with lesions affecting one lobe than in the groups with two to three lobes or four to five lobes, and the counts of B lymphocytes were significantly higher in the group with one lobe and the group with two to three lobes than in the group with four to five lobes. The counts of CD4^+^T and CD8^+^T lymphocytes were highest in the no cavity group and showed a downward trend with the increase in cavities; the T lymphocyte count was significantly higher in the no cavity group than in the group with five or more cavities (*P* = 0.014), and the CD8^+^T lymphocyte count was significantly higher in the no cavity group than in the group with one to two cavities and the group with five or more cavities (*P* = 0.001 and 0.01, respectively).

**Conclusions:**

In most patients with tuberculosis, immune function is impaired. The absolute counts of peripheral blood lymphocyte subsets are closely related to the aetiological results and lesion severity in patients with PTB; this could be used as evidence for immune intervention and monitoring curative effects.

## Graphical abstract

The present study aims to further the understanding of the immune status of patients with TB by using flow cytometry to determine the absolute counts of T lymphocytes, CD4^+^ and CD8^+^T lymphocytes, NK lymphocytes, NKT lymphocytes, and B lymphocytes in 217 cases of pulmonary TB (PTB). The absolute counts of T, CD4^+^T, CD8^+^T, natural killer (NK), NKT, and B lymphocytes in 217 cases of PTB were detected, and the variations in lymphocyte subset counts between different ages and genders and between etiological detection results and chest radiography results were analyzed. The characteristics of lymphocyte subsets in patients with PTB were analyzed and the importance of lymphocyte subset detection in the clinical evaluation and treatment of patients with TB was discussed.

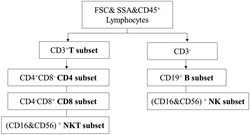

## 1 BACKGROUND

Tuberculosis (TB) is a global disease and a serious threat to human health. According to a recent World Health Organization TB report,^1^ in 2019, there were 10 million cases worldwide; of these, 4500 000 patients had rifampicin‐resistant or multidrug‐resistant TB, and a total of 1.4 million TB‐induced deaths were reported. China is one of 22 countries with a high TB incidence and one of 27 countries with a severe TB resistance.

In recent years, awareness has grown that (1) TB is not only an infectious disease but also an immune disease and (2) *Mycobacterium tuberculosis (M. tb)* interacts with the host's immune system following infection. The occurrence, development and prognosis of TB are closely related to the number and virulence of invasive *M. tb* as well as the immune status and immune response of the host.^2^ Therefore, it is important to evaluate the immunity of the host and his/her clinical immunological characteristics.

However, at present, the only permitted methods for evaluating the immune function of patients with TB in a clinical setting are as follows: (1) humoral immune function analysis, which primarily detects anti‐TB antibodies in sera; (2) cellular immune function analysis, which mainly analyses percentages and absolute counts of lymphocyte subsets; (3) cytokine profiles via interferon‐gamma release assays; and (4) delayed‐type hypersensitivity reactions, such as the tuberculin skin test.

The immune response against TB is principally cell mediated. Therefore, flow cytometry analysis of peripheral blood lymphocyte subsets is an effective method for the clinical evaluation of the immune status, immune function and immune balance of patients with TB; it can provide a basis for immune intervention, curative effect observation and prognosis judgement. This detection method has been widely applied in patients with haematology, cancer or acquired immune deficiency syndrome; however, there are few reports on its application in patients with TB.^3^

According to the expression of cluster of differentiation (CD) antigens on the cell's surface, human peripheral blood lymphocytes can be routinely divided into three subsets: (1) T lymphocytes (CD3^+^); (2) B lymphocytes (CD19^+^); and (3) natural killer (NK) lymphocytes (CD3^−^CD16^+^CD56^+^).

Typically, T cells (of which 90%–95% are αβ T cells) account for >65% of the total lymphocyte count and are the most important immune effector cells in humans. T cells mainly include helper or inducible T cells (CD3^+^CD8^−^CD4^+^, also known as CD4^+^T cells), cytotoxic or inhibitory T cells (CD3^+^CD8^+^CD4^−^, also known as CD8^+^CD4^−^ or CD8^+^T cells) and NKT cells.

Protective cellular immunity against TB is mainly mediated by CD4^+^T lymphocytes, with support from CD8^+^T lymphocytes.^4^, ^5^ The main functions of CD4^+^T cells are the production of cytokines, such as interferon‐γ, and the Th1 response cellular immunity reaction against *M. tb* infection.^4^ Natural killer cells provide innate protection against *M. tb*, and it has been suggested that these cells may facilitate the eradication of intracellular *M. tb* via apoptosis.^6^ Natural killer T cells, which can be identified by the phenotype CD3^+^CD56^+^, have also been shown to participate in TB immunity.^7^ In addition, B lymphocytes assist the anti‐TB cellular immune response by transforming into plasma cells, which secrete anti‐*M. tb* antibodies after activation.^8^

Despite the pivotal role of lymphocytes in anti‐TB immunity, most researchers studying changes in lymphocyte subsets in TB cases have employed the percentage method rather than absolute counts, leading to inconsistent results.^9^, ^10^, ^11^ Furthermore, few reports have utilized large sample sizes; therefore, a systematic and comprehensive understanding of the clinical value of absolute lymphocyte subset counts is lacking at present.

The present study aims to further the understanding of the immune status of patients with TB by using flow cytometry to determine the absolute counts of T lymphocytes, CD4^+^ and CD8^+^T lymphocytes, NK lymphocytes, NKT lymphocytes and B lymphocytes in 217 cases of pulmonary TB (PTB). The characteristics of lymphocyte subsets in patients with PTB were analysed, and the importance of lymphocyte subset detection in the clinical evaluation and treatment of patients with TB was discussed.

## 2 METHODS

### 2.1 Patients

The present study population comprised 217 patients with PTB hospitalized in the Department of Tuberculosis of the Eighth Medical Center, PLA General Hospital, China; the patients were enrolled between April and October 2018.

The primary clinical characteristics of the patients are summarized in Table 1. The diagnostic criteria (including TB history; treatment efficacy; symptoms; indicators; and aetiological, imaging and histopathological examinations) were based on the ‘WS 288–2017 Diagnosis for pulmonary tuberculosis’ and ‘WS 196–2017 Classification of tuberculosis’ standards.^12^, ^13^ All patients were negative for human immunodeficiency virus infection and were not receiving immunosuppressive agents.

**TABLE 1:** Comparisons of lymphocyte subset absolute counts among the different groups divided by clinical parameters in 217 simple PTB patients

Groups	Cases	Mean ± SD of absolute lymphocyte subset counts (cells/μl)						*P*‐value
		T subset	CD4^+^ subset	CD8^+^ subset	NK subset	NKT subset	B subset	
**Age**								
≤25	47	1247 ± 425.1 ^a1^ (73.18 ± 6.69)	675.5 ± 263.5 ^a2^ (39.49 ± 6.67)	482.4 ± 181.5^a3,b3^ (28.52 ± 6.14)	181.4 ± 110.8 (10.52 ± 5.43)	64.2 ± 49.5 (3.89 ± 2.77)	254.5 ± 162.5 ^a4^ (14.26 ± 6.18)	a1, 0.006; b1, 0.000; c1, 0.012 a2, 0.026; b2, 0.000; c2, 0.029; d,0.044 a3, 0.048; b3, 0.015; c3, 0.005 a4, 0.000; b4, 0.000; c4, 0.005
26–44	61	1378 ± 733.2 ^b1,c1^ (71.19 ± 9.09)	803.3 ± 460.4 ^b2,c2^ (41.81 ± 9.65)	502.0 ± 313.1 ^b3c3^ (25.77 ± 6.73)	263.4 ± 242.8 (13.79 ± 8.97)	93.0 ± 97.0 (5.18 ± 6.41)	250.8 ± 148.5 ^b4^ (13.15 ± 6.03)	
45–59	53	1110 ± 553.3 ^c1^ (72.16 ± 9.41)	656.1 ± 376.1 ^c2,d^ (41.95 ± 9.27)	409.3 ± 227.0 ^a3^ (27.02 ± 10.22)	190.5 ± 122.1 (12.96 ± 6.66)	88.4 ± 84.6 (5.67 ± 4.37)	205.5 ± 151.2 ^c4^ (12.91 ± 6.36)	
≥60	56	935.5 ± 450.2^a1,b1^ (70.55 ± 11.10)	517.9 ± 263.1^a2,b2,d^ (39.45 ± 9.42)	388.9 ± 236.5^c3^ (28.76 ± 10.83)	219.6 ± 164.4 (16.80 ± 10.57)	74.1 ± 70.3 (5.47 ± 4.63)	130.1 ± 89.4 ^a4,b4,c4^ (10.14 ± 5.95)	
**Gender**								
Male	136	1211.4 ± 599.1 (71.91 ± 9.69)	690.3 ± 377.5 (40.75 ± 8.99)	459.6 ± 261.0 (27.56 ± 8.83)	223.9 ± 150.4 (14.13 ± 8.88)	90.9 ± 89.5 ^a^ (5.51 ± 5.36)	209.4 ± 152.4 (11.95 ± 6.08)	a, 0.0257
Female	81	1078.1 ± 553.4 (71.21 ± 9.26)	615.3 ± 356.4 (40.75 ± 8.99)	411.4 ± 228.5 (27.34 ± 9.97)	207.2 ± 206.7 (13.43 ± 8.28)	66.1 ± 56.2 ^a^ (4.83 ± 5.23)	206.7 ± 142.9 (13.40 ± 6.71)	
**Aetiological examination**								
Negative	71	1255 ± 78.09 (71.81 ± 9.84)	704.5 ± 46.0 (39.11 ± 8.86)	479.3 ± 34.1 (28.29 ± 7.36)	208.5 ± 16.7 (13.63 ± 9.36)	78.42 ± 8.7 (5.06 ± 3.95)	219.2 ± 17.9 (12.56 ± 5.89)	
Positive	125	1110 ± 47.41 (71.69 ± 8.53)	636.5 ± 31.1 (41.47 ± 8.48)	422.6 ± 20.8 (27.31 ± 10.20)	214.7 ± 13.5 (13.79 ± 7.51)	85.03 ± 7.7 (5.93 ± 6.73)	205.6 ± 13.3 (12.71 ± 6.73)	
**Chest radiography**								
1 lobe	47	1501.0 ± 663.2 ^a1,b1^ (72.76 ± 6.72)	845.7 ± 490.9 ^a2,b2^ (39.50 ± 8.53)	571.9 ± 224.3 ^a3,b3^ (29.00 ± 8.03)	241.4 ± 126.9 (12.36 ± 6.20)	95.9 ± 91.0 (4.90 ± 4.34)	275.2 ± 170.6 ^a4^ (12.83 ± 4.68)	a1, 0.000; b1, 0.002 a2,0.000; b2, 0.016 a3, 0.000; b3, 0.001 a4,0.000; b4, 0.038
2 ~ 3 lobes	59	1165.6 ± 495.5 ^b1^ (70.29 ± 8.47)	675.6 ± 327.5 ^b2^ (40.74 ± 8.36)	416.1 ± 174.5 ^b3^ (25.63 ± 6.84)	233.8 ± 228.7 (14.17 ± 9.21)	75.2 ± 66.0 (4.42 ± 3.44)	222.6 ± 134.4 ^b4^ (13.32 ± 5.12)	
4 ~ 5 lobes	111	1032.5 ± 540.2 ^a1^ (71.98 ± 10.53)	584.9 ± 300.0 ^a2^ (41.25 ± 9.43)	408.4 ± 280.0 ^a3^ (27.75 ± 9.85)	196.8 ± 157.5 (13.94 ± 8.98)	77.3 ± 80.0 (5.53 ± 5.62)	174.5 ± 134.3^a4,b4^ (12.03 ± 7.32)	
**Cavities**								
0	73	1310.5 ± 652.2 ^a1^ (74.50 ± 7.80)	719.9 ± 406.0 (40.32 ± 9.50)	520.9 ± 300.8 ^a2,b^ (30.04 ± 10.01)	199.2 ± 215.0 (10.77 ± 6.66)	229.1 ± 162.2 (4.86 ± 4.48)	1310.5 ± 652.2 (12.73 ± 6.66)	a1, 0.014; a2, 0.001; b, 0.01
1–2	76	1138.5 ± 445.7 (69.89 ± 9.46)	651.6 ± 282.1 (39.97 ± 8.92)	416.5 ± 181.0 ^b^ (26.05 ± 7.56)	243.3 ± 144.6 (15.50 ± 9.09)	200.1 ± 111.4 (4.36 ± 3.00)	1138.5 ± 445.7 (12.11 ± 4.89)	
3–4	24	1089.9 ± 550.3 (70.83 ± 9.94)	606.5 ± 322.7 (40.57 ± 8.29)	457.9 ± 274.3 (28.56 ± 10.04)	202.7 ± 159.4 (13.30 ± 7.01)	231.5 ± 152.1 (5.35 ± 4.16)	1089.9 ± 550.3 (14.37 ± 6.61)	
≥5	44	1035.6 ± 662.4 ^a1^ (70.61 ± 10.07)	633.82 ± 454.0 (42.84 ± 8.33)	365.8 ± 226.0 ^a2^ (24.95 ± 6.67)	206.5 ± 154.5 (15.46 ± 9.81)	180.8 ± 174.0 (6.59 ± 7.48)	1035.6 ± 662.4 (12.05 ± 7.43)	

*Notes*: The contents in parentheses indicate the percentage (%) of the absolute count of the corresponding lymphocyte subsets. In the age group, a1 represents the difference of the counts of T subset between the patients (≥60) and the patients (≤25), b1 represents the difference of the counts of T subset between the patients (≥60) and the patients (26–44), and c1 represents the difference of the counts of T subset between the patients (45–59) and the patients (26–44). a2 represents the difference of the counts of CD4^+^ subset between the patients (≥60) and the patients (≤25), b2 represents the difference of the counts of CD4^+^ subset between the patients (≥60) and the patients (26–44), c2 represents the difference of the counts of CD4 subset between the patients (45–59) and the patients (26–44), and d2 represents the difference of the counts of CD4 subset between the patients (≥60) and the patients (45–59). a3 represents the difference of the counts of CD8 subset between the patients (45–59) and the patients (≤25), b3 represents the difference of the counts of CD8 subset between the patients (≤25) and the patients (26–44), and c3 represents the difference of the counts of CD8 subset between the patients (45–59) and the patients (≥60). a4 represents the difference of the counts of B subset between the patients (≥60) and the patients (≤25), b4 represents the difference of the counts of B subset between the patients (≥60) and the patients (26–44), and c4 represents the difference of the counts of B subset between the patients (45–59) and the patients (≥60). The absolute counts of T, CD4^+^, CD8^+^, NK, NKT and B lymphocytes peaked at age 26–44 and then decreased gradually with an increase in age. This trend was especially notable for T, CD4^+^, CD8^+^ and B lymphocytes; the absolute counts in PTB patients aged over 60 were significantly lower (*P* < 0.05) than those of patients aged 26–44. In the gender group, a represents the difference of the counts of NKT subset between the patients (male) and the patients (female). In the gender group, there was significant difference of the counts of NKT subset between the patients (male) and the patients (female). In the pathogenic examination group, there was no significant difference of the absolute counts of lymphocyte subsets between the two groups. In the chest radiography group, a1 represents the difference of the counts of T subset between the patients (one lobe) and the patients (four to five lobes), and b1 represents the difference of the counts of T subset between the patients (one lobe) and the patients (two to three lobes). a2 represents the difference of the counts of CD4^+^ subset between the patients (one lobe) and the patients (four to five lobes), and b2 represents the difference of the counts of CD4^+^ subset between the patients (one lobe) and the patients (two to three lobes). a3 represents the difference of the counts of CD8^+^ subset between the patients (one lobe) and the patients (four to five lobes), b3 represents the difference of the counts of CD8^+^ subset between the patients (one lobe) and the patients (two to three lobes). a4 represents the difference of the counts of B subset between the patients (one lobe) and the patients (four to five lobes), and b4 represents the difference of the counts of B subset between the patients (two to three lobes) and the patients (four to five lobes). The absolute counts of T, CD4^+^, and CD8^+^ T lymphocytes were higher in the one lobe group than in the other two groups, and the levels of B lymphocytes in the one lobe and two to three lobe groups were significantly higher than in the four to five lobe group; the absolute counts of NK and NKT cells also showed a decreasing trend with the severity of the lesion, but there was no significant difference among the groups. In the cavities group, a1 represents the difference of the counts of T subset between the patients (zero cavity) and the patients (≥5 cavities). a2 represents the difference of the counts of CD8 subset between the patients (zero cavity) and the patients (≥5 cavities), b represents the difference of the counts of CD8 subset between the patients (0) and the patients (one to two cavities). The absolute counts of T, CD4^+^ and CD8^+^ lymphocytes were highest in the no cavity group and showed a downward trend as the number of cavities increased. The absolute count of T lymphocytes in the no cavity group was significantly higher than in the five or more cavities group, and the absolute count of CD8^+^ lymphocytes in the no cavity group was significantly higher than in the one to two cavities group and the five or more cavities group. There was no apparent change among the other four lymphocyte subsets.

### 2.2 Blood sampling

A 2‐mL venous peripheral blood sample was collected from each patient upon hospitalization. The sample was reversed six to eight times in anticoagulant blood vessels for mixing.

### 2.3 Absolute counts of blood lymphocyte subsets determined by flow cytometry

To measure the absolute counts of lymphocyte subsets, 50 μl of the sampled blood was transferred to Becton, Dickinson and Company (BD) Trucount tubes (BD, USA; product code: 340334) containing an aliquot of 20 μ six‐colour T, B and NK lymphocytes (TBNK) reagent, including the following fluorochrome‐labelled monoclonal antibodies: anti‐CD45 (PerCP‐Cy5.5), anti‐CD3 (FITC), anti‐CD4 (PE‐Cy7), anti‐CD8 (APC‐Cy7), anti‐CD16^+^CD56 (PE) and anti‐CD19 (APC) (BD, USA; product code: 337166).

**FIGURE 1:**
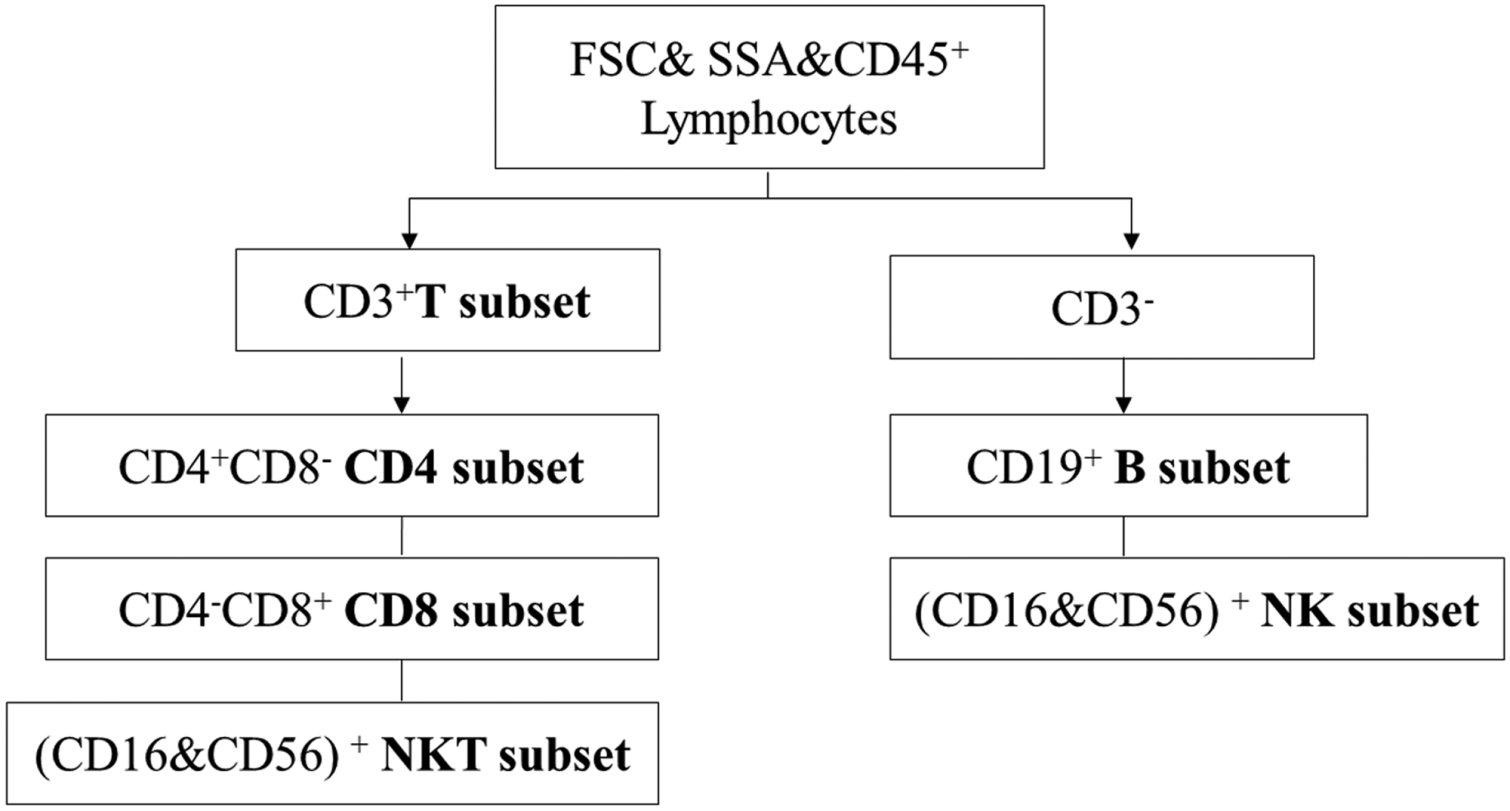
BD six‐colour TBNK reagent including the following fluorochrome‐labelled monoclonal antibodies: anti‐CD45 (PerCP‐Cy5.5), anti‐CD3 (FITC), anti‐CD4 (PE‐Cy7), anti‐CD8 (APC‐Cy7), anti‐CD16 + CD56 (PE), and anti‐CD19 (APC)

The samples were homogenized and incubated in the dark for 15 min at room temperature. Next, the red blood cells in each sample were lysed by adding 450 μl of fluorescence activated cell sorting (FACS) lysis solution (Becton Dickinson, product code: 349202) diluted with ddH_2_O at a ratio of 1:10 and incubated in the dark for 15 min at room temperature. The cells were then analysed using a FACS Arial II flow cytometer (BD, San Jose, CA, USA). The definition of the lymphocyte gate for each of the six subsets is shown in Figure 1.

At least 5000 lymphocytes were obtained, and the software Diva was used for data analysis. The absolute counts for each subset were calculated as follows: cell/L = (total number of cells acquired × total number of beads)/(total number of beads acquired × sample volume).

### 2.4 Patient clinical data collection

The clinical data of 217 cases of simple PTB were retrospectively collected, and the following parameters and information were recorded: patient (1) age and gender; (2) medical history; (3) aetiological detection results of *M. tb* in sputum (including sputum smear, sputum culture and DNA and RNA detection); and (4) X‐ray and computed tomography (CT) results.

The sputum smear was conducted with Ziehl–Neelsen acid‐fast staining; the sputum culture was performed using the BACTEC MGIT 960 mycobacterium culture and detection system (Becton Dickinson Company); DNA detection was carried out using a mycobacterial real‐time polymerase chain reaction detection kit (Capital Bio, Beijing, China); and RNA detection was carried out using the isothermal RNA amplification assay for *M. tb* (REMDU Biotechnology, Shanghai, China) using the technique of simultaneous amplification and testing.

### 2.5 Statistical analysis

The data were managed with Excel 2016 (Microsoft, Redmond, WA, USA) and transferred to the SPSS 16.0 software (SPSS Corp., Chicago, IL) for statistical analysis. All data are presented as mean ± standard deviation. The differences between the groups were analysed using Student's *t*‐test or the *X* ^2^ test where appropriate. A *P*‐value of <0.05 was considered statistically significant. The reference range of normal values of lymphocyte subsets reported by Zhu et al.^14^

## 3 RESULTS

### 3.1 Analysis of lymphocyte subset results

In 217 patients with PTB, the percentages of T, CD4^+^T, CD8^+^T, NK, NKT and B lymphocytes were 5/217 (2.3%), 12/217 (5.5%), 10/217 (4.6%), 46/217 (%), 44/217 (21.2%) and 21/217 (9.7%), respectively, which were lower than the normal reference values. The absolute counts of T, CD4^+^T, CD8^+^T, NK, NKT and B lymphocytes were 90/217 (41.5%), 97/217 (44.7%), 76/217 (35.0%), 85/217 (39.2%), 75/217 (34.6%) and 47/217 (21.7%), respectively, which were lower than the normal reference values. In 75.3% of the patients, one or more of the six subsets were below the reference range. The number of cases in which the absolute count of cell subsets was lower than the normal value was much higher than the number of cases in which the percentage was lower than the normal value.

In 196 cases, the T lymphocyte percentage was within the normal reference value range, and of which there were 78 cases, the T lymphocyte absolute count was lower than the normal reference value. Only in one case with a normal absolute count, the percentage was lower than the normal reference value.

In 173 cases, the CD4^+^T lymphocyte percentage was within the normal reference value range, and of which there were 80 cases, the CD4^+^T lymphocyte absolute count was lower than the normal reference value. Only in three cases with a normal absolute count, the percentage was lower than the normal reference value.

In 198 cases, the CD8^+^T lymphocyte percentage was within the normal reference value range, and of which there were 66 cases, the CD8^+^T lymphocyte absolute count was lower than the normal reference value. Only in one case with a normal absolute count, the percentage was lower than the normal reference value.

In 168 cases, the NK lymphocyte percentage was within the normal reference value range, and of which there were 46 cases, the NK lymphocyte absolute count was lower than the normal reference value. Only in seven cases with a normal absolute count, the percentage was lower than the normal reference value.

In 161 cases, the NKT lymphocyte percentage was within the normal reference value range, and of which there were 36 cases, the NKT lymphocyte absolute count was lower than the normal reference value. Only in six cases with a normal absolute count, the percentage was lower than the normal reference value.

In 160 cases, the B lymphocyte percentage was within the normal reference value range, and of which there were 27 cases, the B lymphocyte absolute count was lower than the normal reference value. Only in two cases with a normal absolute count, the percentage was lower than the normal reference value. Among all the patients included in the present study, 47 had a lower B lymphocyte percentage than the normal reference.

### 3.2 Variation in absolute counts of lymphocyte subsets with different ages and genders in patients with PTB

In 75.3% of patients with PTB, the absolute counts of six lymphocyte subsets were lower than the normal reference range, and 44% of patients showed lower‐than‐normal CD4^+^T lymphocyte levels.

**FIGURE 2:**
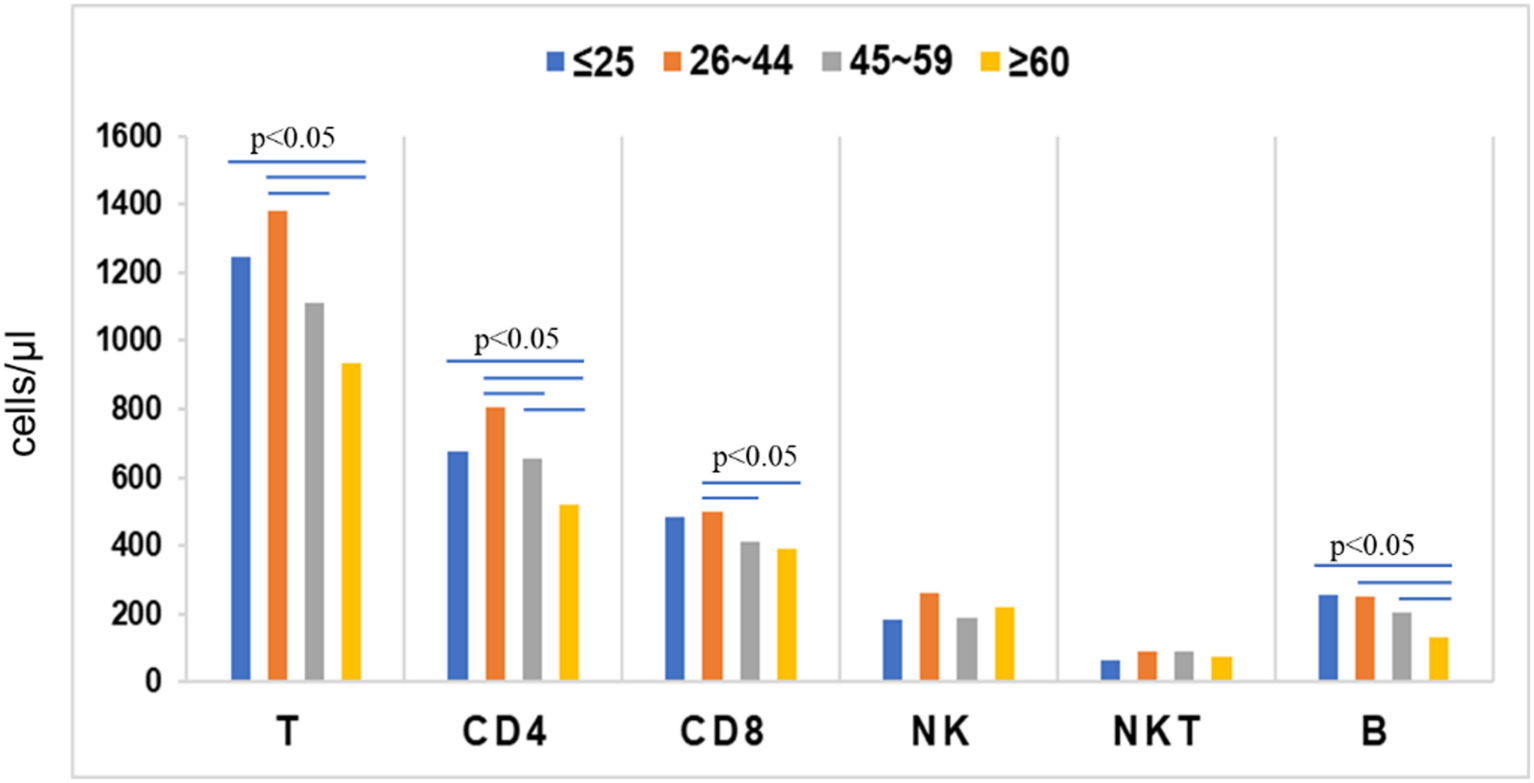
Comparison of lymphocyte subset absolute counts among four age groups of patients with simple PTB. The blue bars indicate significant differences between groups (*P* < 0.05), the Y‐axis represents the mean values of the lymphocyte subset counts (cells/μl), and the X‐axis represents the lymphocyte subsets in the four age groups

**FIGURE 3:**
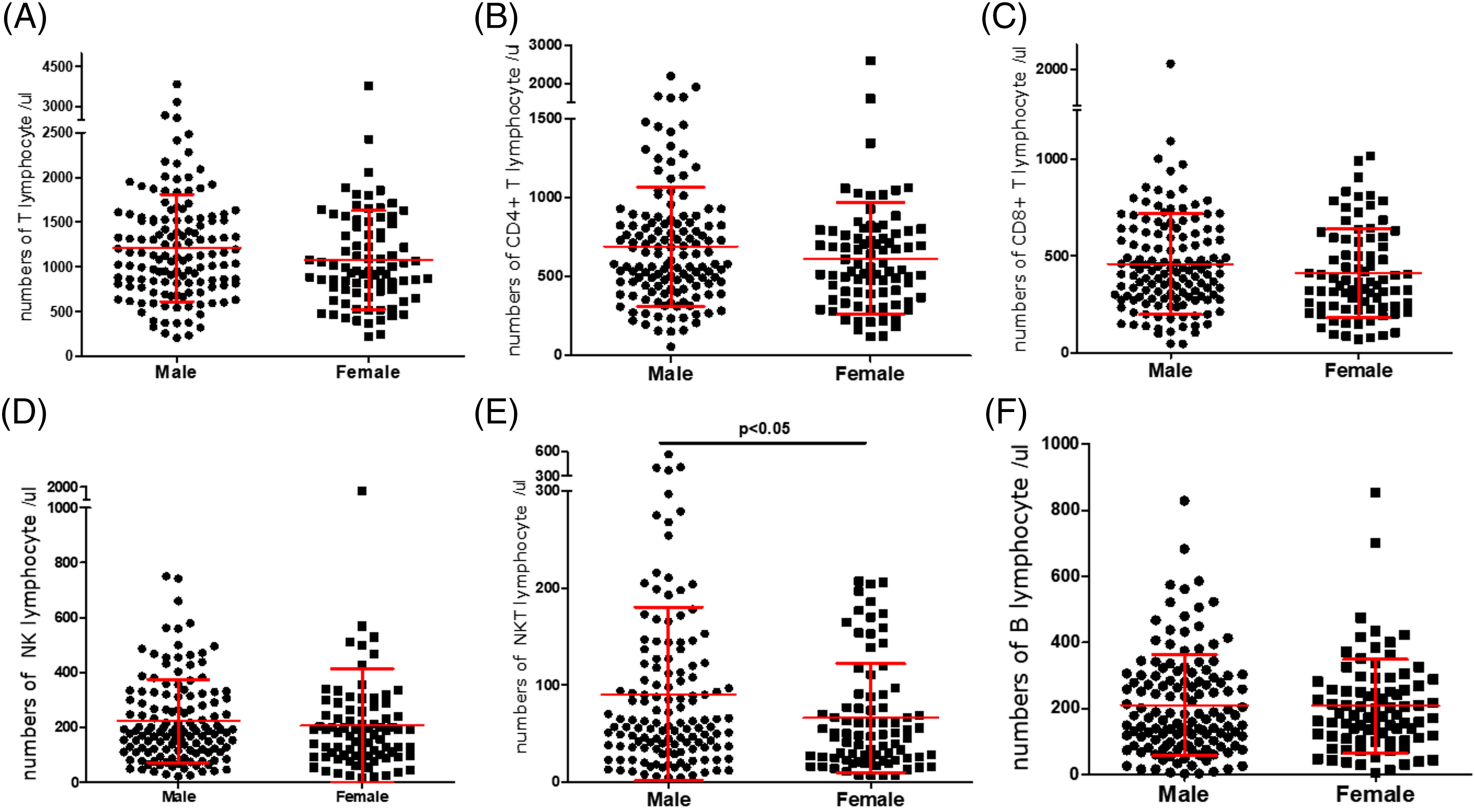
Comparison of lymphocyte subset absolute counts between genders in patients with simple PTB. (A) T lymphocytes (CD3^+^), (B) CD4^+^T lymphocytes (CD3^+^CD8^−^ CD4^+^), (C) CD8^+^T lymphocytes (CD3^+^CD8^+^ CD4^−^), (D) natural killer (NK) cells (CD3^−^CD16^+^CD56^+^), (E) NKT cells (CD3^+^CD16^+^CD56^+^) and (F) B lymphocytes (CD3^−^CD19^+^). The black bar marks the significant difference (*P* < 0.05). The red bars indicate the mean ± standard deviation levels of lymphocyte subset absolute counts in each group

The patients were divided into four age groups: (1) <25 years; (2) 26–44 years; (3) 45–59 years; and (4) >60 years (Figure 2 and Table 1). The absolute counts of T, CD4^+^T, CD8^+^T, NK, NKT and B lymphocytes peaked at age 26–44 and then decreased gradually as the age increased. This trend was especially notable for T, CD4^+^T, CD8^+^T and B lymphocytes; the absolute counts were significantly lower in patients aged >60 years than in patients aged 26–44 years (*P* < 0.05; Table 1). Figure 3 illustrates that the absolute counts of all six subsets were higher in male patients than in female patients; however, this difference was only significant in NK cells (*P* = 0.0257).

### 3.3 Variation in absolute counts of lymphocyte subsets with aetiological detection results in patients with PTB

The aetiological detection results of *M. tb* in sputum included sputum smear, sputum culture and DNA and RNA detection. Patients who received positive results in any of these detection methods were classified as the positive group, and the remaining patients were assigned to the negative group.

Through comparison of the lymphocyte subset absolute counts between the two groups, it was found that there was no significant difference (Table 1). However, the percentage of patients with lower‐than‐normal absolute counts of T, CD4^+^T, CD8^+^T, NK and NKT lymphocytes was higher in the positive group than in the negative group. This difference was significant in the case of CD8^+^T lymphocytes (*P* = 0.0295; Table 2).

**TABLE 2:** Comparisons of cases (%) below the standard range of lymphocyte subset absolute counts^14^ among the different groups divided by clinical parameters in 217 cases simple PTB patients

Groups	Total cases	Cases (%) below the standard range						*P*‐value
		T subset (<955 cells/μl) (<50%)	CD4^+^ subset (<550 cells/μl) (<27%)	CD8^+^ subset (<320 cells/μl) (<15%)	NK subset (<150 cells/μl) (<7%)	NKT subset (<40 cells/μl) (<2%)	B subset (<90 cells/μl) (<5%)	
Aetiological examination								
Negative	71	28(39.4%)	31(43.7%)	18(25.4%) ^a^	26(36.6%)	23(32.4%)	15(21.1%)	a, 0.0295
Positive	125	54(43.2%)	58(46.4%)	51(40.8%) ^a^	49(39.2%)	42(33.6%)	26(20.8%)	

*Notes*: In the chest pathogenic examination group, a represents the difference of the counts of CD8 subset between the patients (negative) and the patients (positive). The percentage of patients with lower‐than‐normal absolute counts of T, CD4^+^, CD8^+^, NK and NKT lymphocytes was higher in the positive group than in the negative group. This difference was significant in the case of CD8 + T lymphocytes (*P* = 0.0295).

### 3.4 Association between absolute counts of lymphocyte subsets and chest radiography results in patients with PTB

The imaging data of patients were observed from two perspectives. First, the patients were divided into three groups according to their lesion lobe numbers: (1) the one lobe group (patients with lesions involving one lobe); (2) the two to three lobe group (patients with lesions involving two to three lobes); and (3) the four to five lobe group (patients with lesions involving four to five lobes).

**FIGURE 4:**
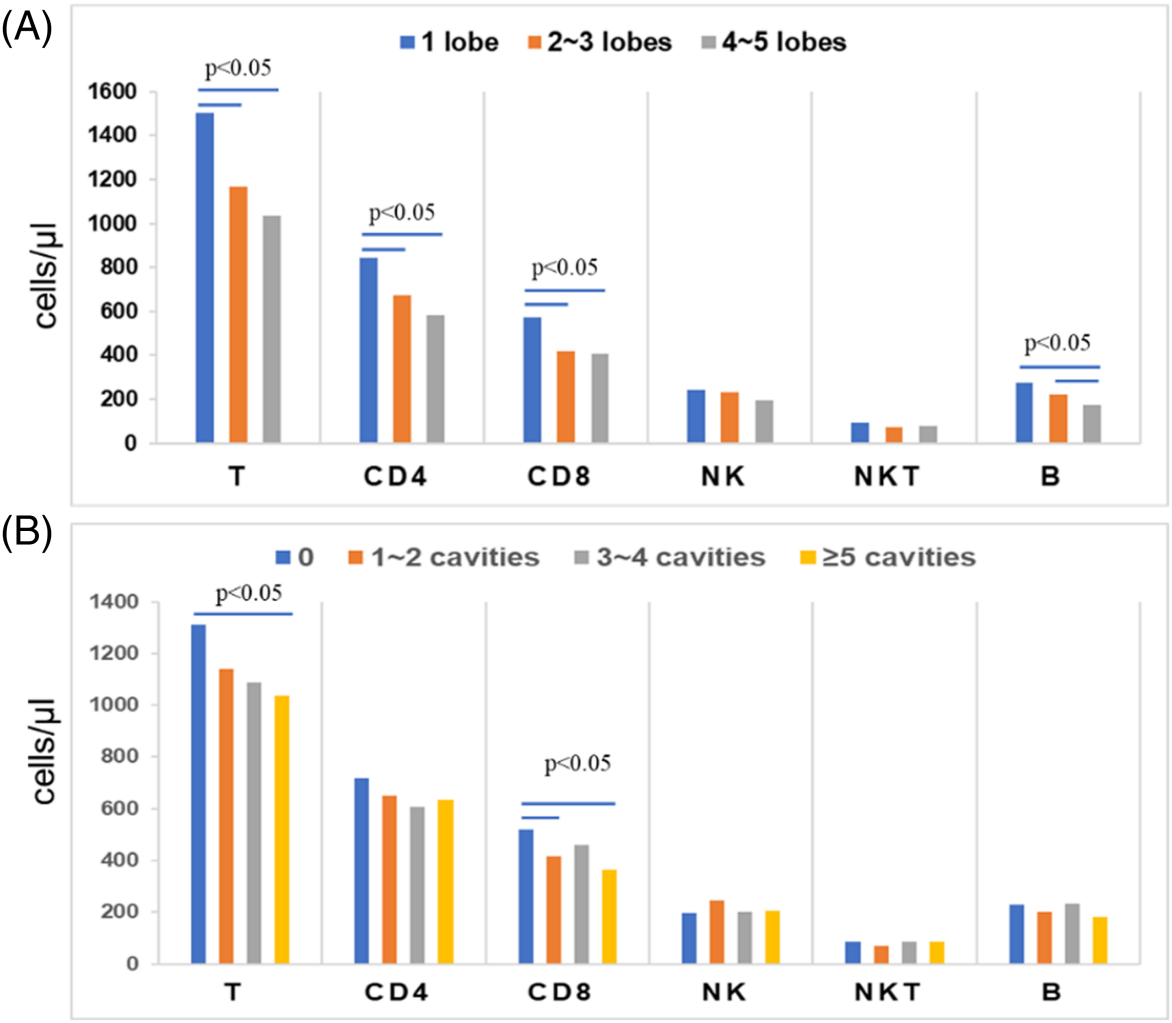
Comparison of lymphocyte subset absolute counts between the different pulmonary lesion lobe groups (A) and the different pulmonary cavity groups (B) in patients with simple PTB. The blue bar marks the significant difference (*P* < 0.05), the Y‐axis represents the mean values (cells/μl) of the lymphocyte subsets, and the X‐axis represents the lymphocyte subset groups

The absolute counts of T, CD4^+^T and CD8^+^T lymphocytes were higher in the one lobe group than in the other two groups, and the levels of B lymphocytes in the one lobe group and two to three lobe group were significantly higher than in the four to five lobe group; the *P*‐values are shown in Table 1. The absolute counts of NK and NKT cells also showed a decreasing trend with the severity of the lesion; however, there was no significant difference among the groups (Figure 4A and Table 1).

The patients were then divided into four groups according to the number of cavities: (1) the no cavity group; (2) the one to two cavity group; (3) the three to four cavity group; and (4) the five or more cavity group.

The absolute counts of T, CD4^+^T and CD8^+^T lymphocytes were highest in the no cavity group and showed a downward trend as the number of cavities increased. The absolute count of T lymphocytes in the no cavity group was significantly higher than in the five or more cavity group (*P* = 0.014), and the absolute count of CD8^+^T lymphocytes in the no cavity group was significantly higher than in the one to two cavity group and the five or more cavity group (*P* = 0.001 and 0.01, respectively). There was no apparent change among the other four lymphocyte subsets (Figure 4B and Table 2).

## 4 DISCUSSION

### 4.1 Lymphocyte profiles in the peripheral blood of patients with PTB

Our study found that the percentage and absolute count of lymphocyte subsets were unequal. Even if the percentage of lymphocyte subsets in patients with PTB was within the normal reference value, the absolute number of lymphocyte subsets was lower than the normal reference value. The absolute number of a lymphocyte subgroup in a small number of patients was within the normal reference value range, but the percentage was lower than the normal reference value. Morais‐Papini et al reported that the absolute numbers of NK, NKT, CD4^+^T and B cells in patients with TB decreased significantly when compared with the control group; furthermore, whereas the percentage of B and NKT cells also decreased, the percentage of CD4^+^T cells increased.^15^ Guglielmetti et al observed a reduction in the absolute numbers of CD4^+^T cells but no difference in the percentage of these cells.^9^ An artificially high percentage may be caused by a reduction in other lymphocyte subsets, and proportions can be maintained in the normal range when all lymphocyte subset counts decrease or increase simultaneously.

The results of this paper, which is based on a large clinical data set, show that the subset counts scarcely exceeded the reference range and that one or more of the six subsets were below the reference range in 75.3% of patients with TB.^16^ This indicates that patients with TB display an altered lymphocyte profile in peripheral blood, creating the need for absolute counts of lymphocyte subsets based on large data in order to draw definite conclusions.

### 4.2 Lymphocyte subsets and clinical features

In the patients observed in the present study, the absolute counts of the six cell subsets peaked at age 26–44 and then decreased as the age increased beyond 45 years. It was found that the absolute counts of the CD4^+^T cell subset were particularly affected by age. The authors of the present study speculate that the disease pathogenesis in young patients with TB (aged <26 years) may be related to an immune cell insufficiency; meanwhile, increased infection opportunities may be responsible for the disease pathogenesis in patients aged 26–44 years.

Immune function decreases in older patients (aged >60 years), where it is manifested by low absolute counts of lymphocyte subsets. Therefore, the authors of this study surmise that age is a key factor affecting the immune status of patients with TB.

The Fifth National TB Epidemiological Sampling Survey in China found that the proportion of elderly patients with TB (aged >60 years) was as high as 48.8%.^17^ Another study found that T and B lymphocyte numbers were significantly decreased in elderly patients with TB compared with younger patients; their immune function was also lower, which directly affected the treatment effect and cure time.^18^

Therefore, the authors of this study suggest that when treating older patients with TB (aged >60 years), clinicians should pay attention not only to drugs that eradicate *M. tb* but also to the immune regulation of patients. For example, giving an immune regulator to improve cellular immune function may be helpful for controlling the disease in older patients.

This study further found that gender factors also appear to impact patient immunity. In the study, the lymphocyte subset counts were higher in male patients than in female patients, especially in NKT cells. This is inconsistent with the fact that TB incidence is significantly higher in males than in females as well as the idea that males may be genetically susceptible to TB.^17^ Therefore, the authors of this study propose that a reduction in lymphocyte count is not the only determinant leading to the development of TB.

Although the average absolute counts of each subset were not significantly affected by aetiological factors in this study, the percentage of patients with TB who had CD8^+^T lymphocyte counts lower than the reference range was noticeably higher in aetiologically positive patients (40.8%) than in aetiologically negative patients (25.4%). This may be due to the fact that CD8^+^T cells play an important part in protective immunity against *M. tb* and can limit pathogen growth via the lysis of *M. tb*‐infected cells.^19^ Therefore, the authors of this study speculate that patients with positive aetiological detection results may have insufficient CD8^+^T cell immune function, which brings challenges to *M. tb* control.

In the present study, disease assessments were carried out using pulmonary X‐ray or CT lesion grading. The absolute counts of T, CD4^+^T, CD8^+^T cells and B lymphocytes decreased significantly with the increase in the number of pulmonary lobes involved, and the absolute counts of T and CD8^+^T cells had the most impact on the presence or absence of cavities. It has previously been reported that the numbers of T, CD4^+^T, CD8^+^T and B lymphocytes were higher in patients with unilateral pulmonary lobe lesions than in patients with bilateral pulmonary lobe lesions.^15^, ^20^ This is consistent with the results of the present study.

Therefore, the authors of this study conclude that reduced lymphocyte counts greatly impact the progression and severity of TB and that lymphocyte subset detection is important for patients with TB who have extensive lesions.

Based on the data obtained from this study, the authors believe that the absolute counts of lymphocyte subset detection is an effective and necessary tool for evaluating the immune status of patients with TB. It can help clinicians make informed judgements, assist in the formulation of patient‐specific treatment plans and help identify patients who would benefit from immune intervention to promote the recovery of immune function.

## 5 CONCLUSION

The absolute counts of T, CD4^+^T, CD8^+^T and B lymphocytes in the peripheral blood of patients with TB decreased with ageing and lesion severity. These results confirm that the immune defence function in most patients with TB is impaired and that it is important to focus on the absolute counts of T lymphocytes and the percentage of T lymphocytes in TB cases.

Because absolute counts of lymphocyte subsets are closely related to both lesion severity and aetiological results, they can be used as a basis for immune intervention and monitoring curative effects. However, lymphocyte subset counts alone cannot adequately meet the clinical needs of patients with TB, and it is necessary to establish new immune function indicators to further guide the clinical diagnosis and treatment of TB.

## CONFLICT OF INTEREST

The authors declare that they have no competing interests.

## AUTHOR CONTRIBUTIONS

Conception and design of the research: Xue‐Qiong Wu. Acquisition of data: Xue‐Juan Bai, Jian‐Qin Liang, Hui‐Ru An, Tao Wang, Zhong‐Yuan Wang, Yong Xue, Yin‐Ping Liu, Lan Wang. Analysis and interpretation of the data: Xue‐Juan Bai. Statistical analysis: Xue‐Juan Bai. Obtaining financing: Xue‐Qiong Wu. Writing of the manuscript: Hui‐Ru An, Xue‐Juan Bai, Jian‐Qin Liang. Critical revision of the manuscript for intellectual content: Xue‐Qiong Wu. All authors read and approved the final draft.

## ETHICS STATEMENT

The study was conducted in accordance with the Declaration of Helsinki (as was revised in 2013). The study was approved by Ethics Committee of the Eighth Medical Center of PLA General Hospital in China. The signed informed consent was obtained from all participants before the investigation.

## Data Availability

The data that support the findings of this study are available from the corresponding author upon reasonable request.
